# Immune Checkpoint Inhibitors for Advanced Hepatocellular Carcinoma: Monotherapies and Combined Therapies

**DOI:** 10.3389/fonc.2022.898964

**Published:** 2022-06-16

**Authors:** Tao Ouyang, Xuefeng Kan, Chuansheng Zheng

**Affiliations:** ^1^ Department of Radiology, Union Hospital, Tongji Medical College, Huazhong University of Science and Technology, Wuhan, China; ^2^ Hubei Province Key Laboratory of Molecular Imaging, Wuhan, China

**Keywords:** monotherapy, combination therapy, hepatocellular carcinoma, immune, PD-1

## Abstract

Hepatocellular carcinoma (HCC) is an important cause of cancer death and is considered the 3rd most lethal around the world. Hepatectomy, liver transplantation, and ablation therapy are considered curative treatments for early-stage HCC. Transarterial chemoembolization is the preferred therapy for intermediate stage HCC. Ssystemic therapy is recommended for advanced HCC. For more than a decade, sorafenib and lenvatinib were used as the first-line treatment for the advanced HCC. For the great success of immunotherapy in melanoma and lung cancer, some immune-based treatments, such as immune checkpoint inhibitors (ICIs), have been applied in the treatment of HCC. The anti-programmed cell death protein 1 (PD1) antibodies, including nivolumab and pembrolizumab, have been approved by the Food and Drug Administration for sorafenib-pretreated patients. Moreover, due to the results of durable antitumor responses attained from the phase 3 trials, atezolizumab in combination with bevacizumab is now the standard therapy for advanced HCC. Recently, there are a lot of clinical trials involving the ICIs, as monotherapy or combination therapy, with tyrosine kinase inhibitors, antiangiogenic drugs, cytotoxic agents, and locoregional treatments, providing a promising outcome for advanced HCC. Thus, this review summarized the role of ICIs for HCC patients with monotherapy or combination therapy. The success and failures of monotherapy and combination therapy involving ICIs have provided advanced insights into HCC treatment and led to novel avenues to improve therapy efficacy in HCC.

## Introduction

Hepatocellular carcinoma (HCC) accounts for more than 90% of primary liver cancers and is the third leading cause of cancer-related lethal (1). Based on the Barcelona Clinic Liver Cancer staging (2), hepatectomy, liver transplantation, and ablation therapy were the curative therapies for early-stage HCC. Transarterial chemoembolization (TACE) is the standard treatment for intermediate-stage HCC, and systemic therapy was recommended for advanced HCC (3). Molecular targeted agents played an crucial role in the systemic therapy of advanced HCC. In 2007, based on the results of SHARP trial (NCT00105443) (4) and ORIENTAL trial (NCT00492752) (5), sorafenib, an small molecule multikinase inhibitor, was approved as the preferred therapy for unresectable HCC. Since that, great effort has been made in the research of novel targeted therapy drugs. In 2017, for the promising outcomes of RESORCE study (NCT01774344) (6), regorafenib, a small molecule multitarget inhibitor, was approved for second-line treatment of HCC. Furthermore, some clinical studies for advanced HCC obtained promising outcomes, resulting in the approval of lenvatinib (7) as preferred therapy, and cabozantinib (8) and ramucirumab (9) as second-line therapy for advanced HCC. However, these targeted agents generally have the characteristics of a lower response rate, a high treatment resistance, and frequent adverse events in the systemic therapy for advanced HCC patients (10).

Immune checkpoint inhibitors (ICIs), including the programmed death-1 (PD-1) and the programmed death-ligand 1 (PD-L1), have exhibited potential therapeutic effects for advanced HCC (11–13). Based on the survival efficacy from the results of phase II clinical trials, anti-PD1 inhibitors nivolumab and pembrolizumab were approved as the subsequent-line treatment for unresectable HCC (14, 15). However, the overall response rate (ORR) of nivolumab or pembrolizumab for advanced HCC was 15-20% (16). In order to improve the therapeutic effect of ICIs for HCC, a large number of register clinical trials on the combination treatment with ICIs are being carried out. In 2020, with the positive results obtained from the IMbrave150 study (NCT03434379) (17), atezolizumab (PD-L1 inhibitors) combined with bevacizumab (anti-VEGF agent) has been approved as the preferred therapy for advanced HCC.

To date, numerous clinical trials evaluating the therapeutic effects of ICIs for advanced HCC, including monotherapy and combination therapy. Therefore, in this review, we summarized the development and progress of immune checkpoint-based therapy for advanced HCC based on the completed and ongoing clinical trials around the world, and pointed out the possible future directions for the development of HCC therapeutic drugs.

## Immune Checkpoint Inhibitors for HCC

The liver continuous exposed to over-stimulation, including hepatitis B virus or hepatitis C virus, aflatoxins, and alcohol, which can lead to pathological inflammation, immune system disorders, and destruction of tissue homeostasis, resulting in liver fibrosis, cirrhosis, and even development of cancer (18). HCC is a typical inflammation-related cancer, and the chronic inflammation enhances the tumor immunogenicity and evades the host immune surveillance (19–21). A series of cytokines, chemokines, and growth factors involved in the occurrence and progression of HCC, and the IL-6, IL-1β, and TGF-β are the major cytokines (22, 23). In addition, hepatic sinusoidal endothelial cells (LSECs) and regulatory T cell (Treg) release high levels of immunosuppressive cytokines, resulting in an immunosuppressive microenvironment of the liver, and a large number of immune checkpoint molecules expressed on immune cells (24–28).

**Figure 1:**
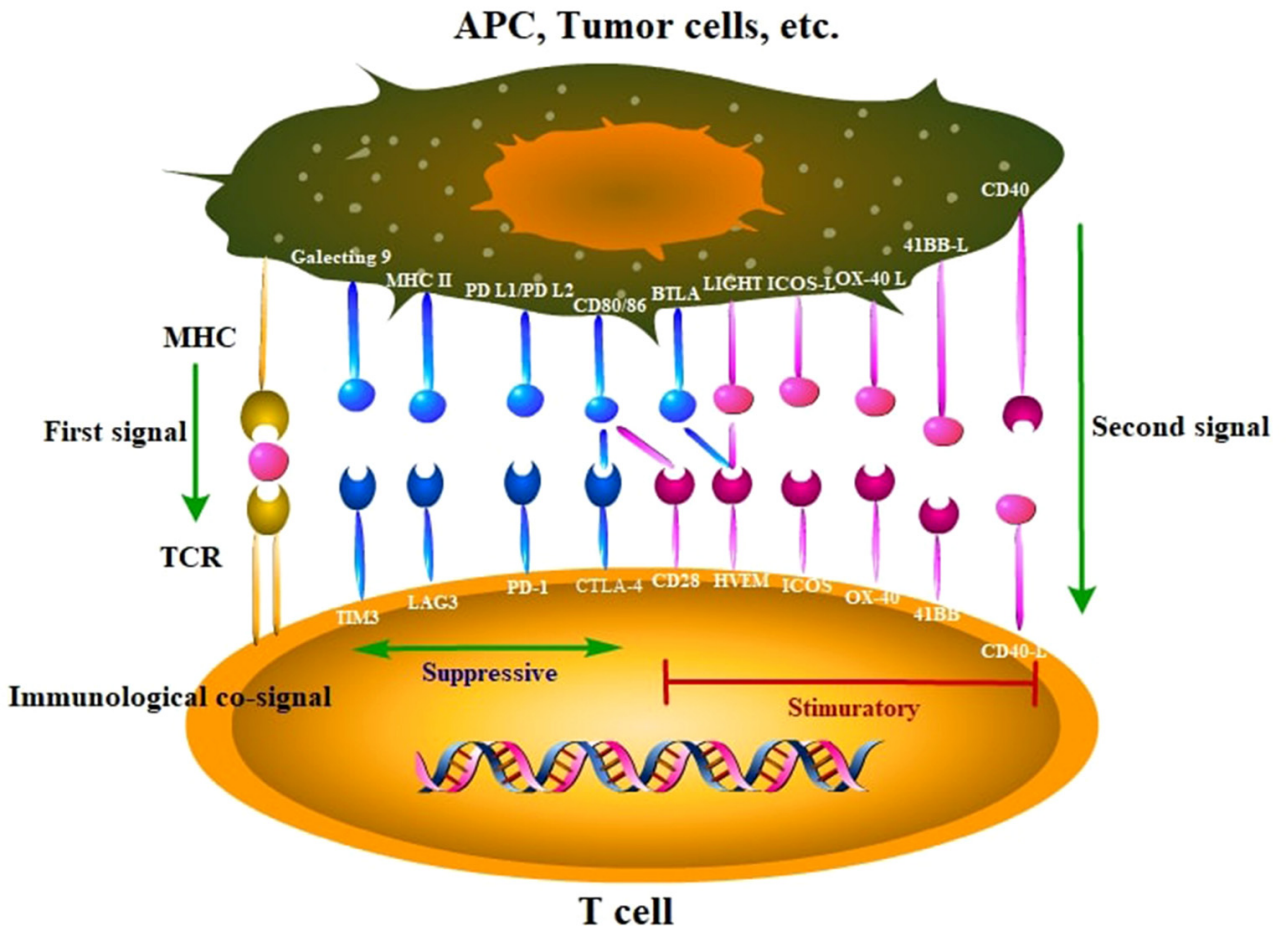
Main immune molecules and corresponding receptors that suppressive or stimulatory immune responses. These ligands and cognate receptors expressed in tumor cells and immune cells are known as immune checkpoints. APC, antigen presenting cell; MHC, major histocompatibility complex; TCR, T-cell receptor.

**Figure 2:**
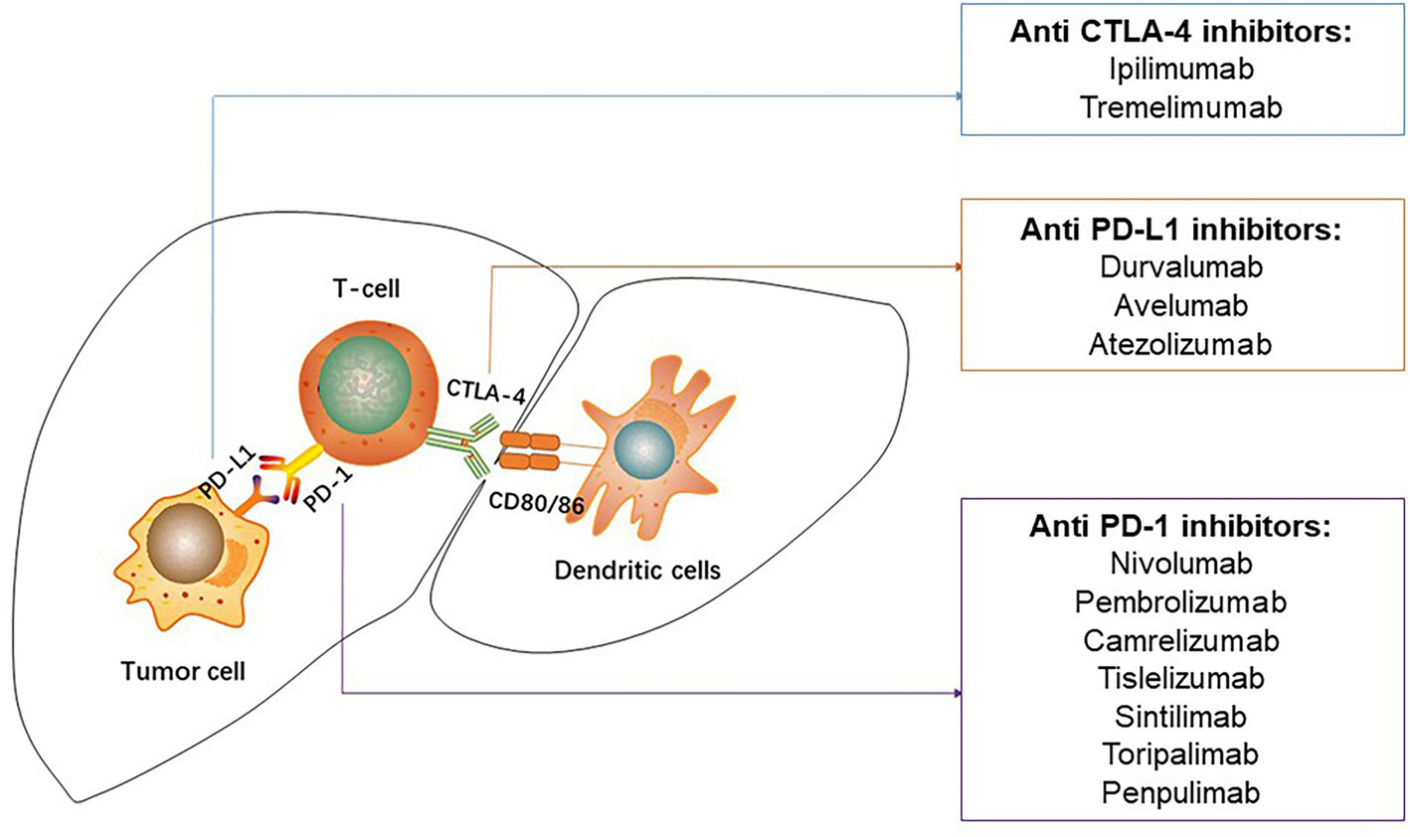
Immune checkpoint inhibitors used in hepatocellular carcinoma. PD-1, programmed death-1; PD-L1, programmed death-ligand 1; CTLA-4, cytotoxic T-lymphocyte associated protein-4.

Immune checkpoints are intrinsic to the immune system to prevent autoreactivity (**Figure 1**). PD-1, a co-inhibitory receptor molecule, which is mainly expressed in CD4^+^ T cells or antigen presenting cells, plays a vital role in regulating peripheral immune tolerance (29–31). PD-L1 is overexpressed on dendritic cells, macrophages, and liver parenchymal cells (32). The interaction of PD-1 and PD-L1 is one of the important mechanisms of tumor immune escape (33, 34). Moreover, the immune inhibitory molecule CTLA-4 are greatly expressed on highly activated Treg cells in the livers (35). The use of immune checkpoint inhibitors is a promising therapeutic to promote immunotherapy in the treatment of cancer, which could block immune checkpoint molecules and reactivate immune responses in the tumor microenvironment (36). To date, the approved ICIs in the treatment of HCC included the anti-PD1 inhibitors nivolumab and pembrolizumab, the anti-PDL1 inhibitor atezolizumab, and the anti-CTLA-4 ipilimumab (**Figure 2**). The monotherapy (**Table 1**) or combination therapy (**Table 2**) involving ICIs for HCC have made a clinical breakthrough.

**Table 1:** Clinical trials of monotherapy of immune checkpoint inhibitors for advanced HCC.

Trial	Drug	Phase	Patients	ORR	mPFS	mOS	TRAEs
NCT01658878	Nivolumab	I/II	262	20%	4.0	NA	NA
NCT02576509	Nivolumab	III	743	15%	NA	16.4	NA
NCT02702414	Pembrolizumab	II	104	17%	4.9	12.9	73%
NCT02702401	Pembrolizumab	III	413	18.3	3.0	13.9	52.7%
NCT02989922	Camrelizumab	II	217	14.7%	NA	NA	67%
NCT01693562	Durvalumab	I/II	104	10.6%	2.1	13.6	60.4%
NCT01008358	Tremelimumab	II	20	17.6	6.5	8.2	65%

ORR, objective response rate; PFS, progressed-free survival; OS, overall survival; m, median; TRAEs, treatment-related adverse events. NA, not available.

**Table 2:** Clinical trials of combined therapies based on immune checkpoint inhibitors for advanced HCC.

Trial	Drug	Phase	Patients	ORR	mPFS	mOS	TRAEs
NCT01658878	Nivolumab + Ipilimumab	I/II	148	29.1%	NA	22.8	80.4%
NCT02715531	Atezolizumab + Bevacizumab	Ib	104	35.6%	5.6	17.1	44%
NCT03434379	Atezolizumab + Bevacizumab	III	336	27.3%	6.8	NA	98.2%
NCT03006926	Pembrolizumab + Lenvatinib	Ib	104	46.0%	9.3	22	67%
NCT03418922	Nivolumab + Lenvatinib	Ib	30	76.7%	NA	NA	100%
NCT01658878	Nivolumab + Cabozantinib	I/II	36	17%	5.5	NA	42%
NCT01853618	Tremelimumab + Ablation	I/II	32	NA	7.4	12.3	NA
NCT03033446	Nivolumab + Radiotherapy	II	36	31%	4.6	15.1	NA
NCT03092895	Camrelizumab + Oxaliplatin	II	34	26.5%	5.5	NA	85.3%

ORR, objective response rate; PFS, progressed-free survival; OS, overall survival; m, median; TRAEs, treatment-related adverse events. NA, not available.

Except for PD-1 and CTLA-4, many coreceptors activate lymphocytes by regulating the antigen receptor signaling to optimize tumor immune responses. Lymphocyte activation gene-3 (LAG-3) is one of the most important targets in these coreceptors (37, 38). LAG-3 expressed on the CD4^+^ and CD8^+^ T cells under the stimulation of antigen (39). The T cells lose effector ability with the continued and high expression of LAG-3 inhibitory coreceptors. To date, LAG-3 inhibitors as monotherapies or in combination with PD-1/PD- L1 inhibitors are conducting clinical trials in multiple cancers (40).

## Monotherapy of Immune Checkpoint Inhibitors

### Nivolumab (Anti-PD1)

Nivolumab is the first fully humanized immunoglobulin G4 (IgG4) anti-PD1 monoclonal inhibitor, and the first ICIs that was approved for the HCC treatment (41). Nivolumab prevents the interaction of the PD-1 with its ligands PD-L1 by binding to PD-1, which can inhibit the immune suppression and immune escape, enhance the activity and proliferation of host T cells, and strengthen the ability of anti-tumor immune response in the tumor microenvironment (42). The superior survival result of nivolumab for the therapeutic of advanced HCC was firstly revealed in the multicenter, phase I/II, open-labeled CHECKMATE-040 trial (14). This is a dose-escalation and expansion study performed in advanced adult HCC patients with or without sorafenib pretreated. Drug dosage was given 0.1–10 mg/kg in the dose-escalation group and 3 mg/kg every 2 weeks in the dose-expansion group. Finally, 262 eligible HCC patients were included. The objective response rate (ORR) was 20% (95% CI: 15%–26%) in the dose-expansion group and 15% (95% CI: 6%–28%) in the dose-escalation group. Meanwhile, in the dose-escalation group, 12 patients suffered from grade 3/4 treatment-related adverse events (TRAEs) and 3 patients experienced treatment-related serious AEs (14).

Then, a phase 3 trial (CheckMate 459) compared the nivolumab with the sorafenib as the primary therapy for HCC patients (43). This study recruited 743 eligible patients, who were evenly randomly allocated to the nivolumab group and the sorafenib group. Nivolumab was given 240 mg every two weeks, and sorafenib was given 400 mg twice a day. Although the median overall survival (OS) was 16.4 months in the nivolumab group, which was significantly longer than 14.7 months in the sorafenib group, it did not reach the pre-defined statistical significance threshold (HR: 0.84, *P* = 0.0419). The anti-tumor efficacy of nivolumab is not better than that of sorafenib for advanced HCC based on the CheckMate 459 results. Thus, it has not been approved for HCC as preferred treatment.

### Pembrolizumab (Anti-PD1)

Pembrolizumab is another IgG4 anti-PD1 inhibitor, and it was approved as the second-line systemic therapy for advanced HCC patients, according to the results of KEYNOTE-224 (15). The multicenter, phase 2 study included 104 eligible pathologically confirmed advanced HCC patients who were intolerant or progresses with sorafenib. Pembrolizumab was given 200 mg every 3 weeks durable 2 years or until disease progression. Eventually, the ORR was 17% (95% CI: 11%–26%). The median progression-free survival (PFS) was 4.9 months (95% CI: 3.4-7.2 months) and the median OS was 12.9 months (95% CI: 9.7-15.5 months). Any grade TRAEs was 73%, which included 24% grade 3 TRAEs.

Subsequently, a randomized, double-blind, phase 3 trial was conducted to compare the efficacy and safety of pembrolizumab with placebo in the treatment of advance HCC patients who previously received sorafenib (KEYNOTE-240) (44). Two hundred and seventy-eight patients received 200 mg pembrolizumab intravenously every 3 weeks durable about 2 years, and 135 patients received saline placebo. At the cutoff date, the median OS was 13.9 months (95% CI: 11.6-16.0 months) in the pembrolizumab group, and 10.6 months (95% CI: 8.3-13.5 months) in the placebo group (*P* = 0.024). The median PFS was 3.0 months (95% CI: 2.8-4.1months) in the pembrolizumab arm, and 2.8 months (95% CI: 1.6-3.0 months) in the placebo arm (*P* = 0.019). However, the primary endpoint of OS and PFS did not reach the prespecified boundaries of statistical significance. In addition, two phase 3 trials involving the monotherapy of pembrolizumab are currently ongoing (KEYNOTE-394 and KEYNOTE-937).

### Camrelizumab (Anti-PD1)

Camrelizumab is a fully humanized anti-PD1 inhibitor, and the binding epitope is different from that of nivolumab and pembrolizumab (45). NCT02989922 trials was a multicenter, open-label, phase 2 single-arm study to assess the efficacy of camrelizumab for patients who were developed or intolerant to previous systemic drugs. In this study, 109 eligible participants received 3 mg/kg intravenously every 2 weeks, and 108 patients received 3 mg/kg intravenously every 3 weeks. Finally, the ORR of camrelizumab was 14.7% (95% CI: 10.3%-20.2%), and the 6-month OS rate was 74.4% (95% CI: 68.0%-79.9%). Meanwhile, grade 3/4 TRAEs was 22%, and the treatment-related death was 0.9% (45).

### Durvalumab (Anti-PD-L1)

Durvalumab is a humanized IgG1 anti-PD-L1 monoclonal antibody (46). It plays the anti-tumor efficacy through binding to the PD-L1 receptor on the surface of cancer cells rather than the PD-1 receptor. NCT01693562 was a multicenter, open-label, phase 1/2 study to assess the clinical efficacy of durvalumab as monotherapy for HCC patients (47). Forty participants were given durvalumab 10 mg/kg intravenously every 2 weeks durable one year or until progressed. The results demonstrated that the ORR was 10.3%, and the median OS was 13.2 months (95% CI: 6.3–21.1 months). This study confirmed the potential clinical efficacy of durvalumab as the second-line therapy for HCC.

### Tremelimumab (Anti-CTLA-4)

Tremelimumab is a humanized IgG2 monoclonal antibody that blocks the binding of CTLA-4, an extracellular inhibitory receptor expressed on T cells (48). CTLA-4 is a CD28 homolog, binding to B7 ligand on antigen-presenting cells, interferes with T cell activation and proliferation. An open-label, pilot phase 2 clinical study firstly evaluated the treatment effect of tremelimumab forHCC and chronic hepatitis C virus (HCV) infection (49). Twenty-one participants received tremelimumab 15 mg/kg intravenously every 3 months until disease development or intolerable adverse event. Finally, the study performed that the disease control rate was 76.4%, the median time to progression (TTP) was 6.48 months (95% CI: 3.95–9.14 months), and the median OS was 8.2 months (95% CI: 4.64–21.34 months). This promising results encouraged more future studies of CTLA-4 inhibitors for advanced HCC.

## Combination Therapy With Immune Checkpoint Inhibitors for Advanced HCC

### Combinations of the Two ICIs

Combinations of two ICIs, such as anti-PD-1 with anti-CTLA-4 inhibitors have been performed in numerous cancers, including HCC. The CheckMate 040 was a clinical trial to evaluate the effect of nivolumab (anti-PD1 agents) plus ipilimumab (anti-CTLA-4 agents) for advanced HCC patients pretreated sorafenib (50). In this study, 148 eligible participants were randomized 1:1:1 to three groups: group A was given at a dosage of nivolumab 1 mg/kg intravenously and ipilimumab 3 mg/kg intravenously, group B was given at a dosage of nivolumab 3 mg/kg intravenously and ipilimumab 1 mg/kg intravenously, and group C was given at a dosage of nivolumab 3 mg/kg intravenously and ipilimumab 1 mg/kg intravenously. The results showed that the ORR was 32% (95% CI: 20%-47%) in the group A, 27% (95% CI: 15%-41%) in the group B, and 29% (95% CI: 17%-43%) in the group C. Moreover, the median OS was 22.8 months in the group A, 12.5 months in the group B, 12.7 months in the group C. Based on these results, in 2020, the combination of nivolumab with ipilimumab was approved as the second-line treatment for advanced HCC patients who failed to sorafenib treatment (50).

### Combination of ICIs With Angiogenesis Inhibitors

Vascular endothelial growth factor (VEGF) overexpression is one of the critical mechanisms of HCC tumor angiogenesis, and it is associated with immunosuppressive effects in the tumor microenvironment (51). VEGF inhibitors alleviate VEGF-mediated immunosuppression in tumors and the tumor microenvironment and promote infiltration of immune cells in tumors. Therefore, the combination therapy of ICIs with VEGF inhibitors may have a synergistic anti-tumor effect for advanced HCC. Atezolizumab is a humanized IgG1 monoclonal antibody, which selectively targets PD­L1, and bevacizumab is a humanized IgG1 monoclonal antibody that targets VEGF (52, 53). A multicenter, open-label, phase 1b clinical trial (NCT02715531) evaluated the effectiveness of atezolizumab (anti-PD-L1 agent) combined with bevacizumab (anti-VEGF agent) in the treatment of HCC (54). In this study, the combination therapy group received atezolizumab 1200 mg intravenously and bevacizumab 15 mg/kg intravenously every 3 weeks until the disease progressed or intolerable adverse events. The results revealed that the ORR was 36% (95% CI: 26%-46%), the median OS was 17.1 months, and the median PFS was 5.6 months (95% CI: 3.6-7.4 months) in the combination therapy group, which significantly longer than that of in the atezolizumab monotherapy group (*P* = 0.011).

Subsequently, a global, phase 3 study (NCT03434379) was performed to compare the combination of atezolizumab with bevacizumab to sorafenib for HCC patients who never received systemic therapy (17). The eligible participants enrolled in this study were randomly assigned in a 2:1 ratio to receive either combined therapy of atezolizumab with bevacizumab or sorafenib alone therapy until the disease progressed or intolerable adverse events. Finally, a total of 336 participants received atezolizumab plus bevacizumab, and 165 patients received sorafenib alone treatment. The survival rates of 6 months (84.8%) and 12 months (67.2%) in the combined therapy group were significantly longer than that of the sorafenib alone group (*P* < 0.001). Moreover, the median PFS was 6.8 months (95% CI: 5.7-8.3 months) in the combination group, which were significantly longer than 4.3 months (95% CI: 4.0-5.6 months) in the sorafenib group (*P* < 0.001). The ORR of the atezolizumab–bevacizumab group was 27.3% (95% CI: 22.5%-32.5%), which was significantly higher than 11.9% (95% CI: 7.4%-18.0%) in the sorafenib group (*P* < 0.001). Based on the encouraging results from this trial, the combination treatment of atezolizumab with bevacizumab was approved as the preferred therapy for the advanced HCC.

Lenvatinib, a multi-kinase inhibitor of VEGF receptors, was permitted for standard therapy of unresectable HCC on the basis of REFLECT study results (7). A phase 1b trial was performed to assess the efficacy of lenvatinib combining with pembrolizumab for the treatment of Barcelona Clinic Liver Cancer (BCLC) stage B or C HCC (55). One hundred and four eligible participants received lenvatinib 12 mg/day or 8 mg/day orally and pembrolizumab 200 mg intravenously every 21 days. The results suggested that the ORR was 46.0% (95% CI: 36.0%-56.3%), and the median PFS was 9.3 months (95% CI: 5.6-9.7 months). According to the encouraging results of this study, the combination therapy of lenvatinib and pembrolizumab is defined as a breakthrough therapy designation in the first-line treatment of HCC. Therefore, a phase 3 trial comparing lenvatinib plus pembrolizumab with lenvatinib plus placebo as standard treatment for advanced HCC is underway. Additionally, the clinical effect of combination lenvatinib with nivolumab for unresectable HCC was investigated in an open-label, phase Ib trial (NCT03418922) (56). The results of this study showed the ORR was 76.7% with tolerable toxicity.

Cabozantinib is a small molecule tyrosine kinase inhibitor that can inhibit the phosphorylation of MET and VEGF receptor 2 in HCC (57). In a randomized, double-blind, phase 3 trial (NCT01908426), HCC patients treated with cabozantinib resulted in a significantly longer OS (median OS: 10.2 months versus 8.0 months) and PFS (median PFS: 5.2 months versus 1.9 months) than that of placebo. Thus, it was approved as the second-line therapy for advanced HCC (8). Recently, the combination of cabozantinib and ICIs showed promising results in the expansion arm from the CheckMate 040 study. In this study, 35 patients received the triple combination therapy of nivolumab, ipilimumab, and cabozantinib, and 36 patients received the doublet combination of nivolumab and cabozantinib. As the researchers envisioned, the ORR in triple combination arm was 26%, which was significantly higher than 17% in the doublet combination arm (*P* < 0.001), and the median PFS in the triple combination arm was significantly longer than that of in the doublet combination arm (6.8 months versus 5.5 months, *P* < 0.001) (58). The combination of cabozantinib with other ICIs, such as pembrolizumab (NCT04442581) and durvalumab (NCT03539822), is in progress.

### Combination of ICIs With Locoregional Therapies

For unresectable HCC, numerous locoregional therapies, including TACE, RFA or microwave ablation, and radiation therapy, are always the limited first-line treatment option (59). Local treatment of HCC can improve anti-tumor immunity by releasing inflammatory factors and tumor-specific neoantigens from killing tumor cells (60, 61). Currently, some clinical trials are investigating the efficacy and safety of the combination of locoregional therapies with immunotherapeutic agents, such as ICIs. A non-randomized, phase 1/2, single-arm study involving 32 patients evaluated the safely and feasibly of tremelimumab combining with ablation for unresectable HCC (62). In this study, patients received tremelimumab at two dose levels (3.5 mg/kg and 10 mg/kg i.v.) every 4 weeks for 6 doses, followed by 3-monthly infusions until off-treatment criteria were met. On day 36, subsequently radiofrequency ablation or chemoablation was conducted. The results showed that the median TTP and OS were 7.4 months (95% CI: 4.7-19.4 months) and 12.3 months (95% CI: 9.3-15.4 months), respectively. Meanwhile, 12 of 14 patients with quantifiable HCV showed a significant reduction in viral load. In addition, a phase 2 study assessed the impact of ablation on unresectable HCC patients who had stable disease or atypical response to nivolumab or pembrolizumab inhibitor after resistance to sorafenib. Additional ablation treatment increased the response rate from 10% to 24% (12/50) with tolerable adverse events. Furthermore, the median TTP, PFS, and OS were 6.1 months (95% CI: 2.6-11.2 months), 5 months (95%CI: 2.9-7.1 months) and 16.9 months (95%CI: 7.7-26.1 months), respectively. Recently, the results of a phase II, non-randomized trial (NCT03033446) revealed that the combination of nivolumab with Y90-radioembolization (RE) was a promising option for Child-Pugh A advanced HCC patients (63). Among the evaluable 36 participants, the DCR was 58.3%, and the median PFS and OS were 4.6 months (95% CI: 2.3-8.4 months) and 15.1 months (95% CI: 7.8-unreached), respectively. Moreover, several clinical trials of drug eluting bead transarterial chemoembolization (DEB-TACE) in combination with nivolumab (NCT03143270), TACE in combination with bevacizumab and durvalumab (NCT03778957), and TACE in combination with pembrolizumab (NCT03397654) are currently underway (64).

### Combination of ICIs With Chemotherapies

Several previous studies suggested that chemotherapy agents can improve anti-tumor immune response and induce immunogenic cell death by activating the dendritic cells, enhancing cross-priming of T cells, and downregulating of myeloid-derived suppressor cells and Treg cells (65, 66). A multicenter, phase 2, single-arm study evaluated the effectiveness of combination of camrelizumab with oxaliplatin-based chemotherapy for advanced HCC patients (67). In the study, 34 eligible patients received camrelizumab (3 mg/kg i.v., every 2 weeks) and typical FOLFOX4 (infusional fluorouracil, leucovorin and oxaliplatin) or GEMOX (gemcitabine and oxaliplatin) regimen. The results showed that the confirmed ORR was 26.5% and DCR was 79.4%. Median time to response (TTR) was 2.0 months. Furthermore, a phase 3 study that compares the combination therapy of camrelizumab with FOLFOX4 regimen to placebo with FOLFOX4 regimen for advanced HCC patients are currently ongoing (NCT03605706).

## Current Challenges

To date, there are still many limitations and challenges in the application of immunotherapy for advanced HCC based on ICIs. First of all, the approved treatment, including monotherapy and combination therapy, for advanced HCC still have limited survival efficacy. Although the combination of ICIs with VEGF inhibitors improved the response rate and survival time, more than two-thirds of the patients still do not respond (68). In addition, some previous studies (14, 15) evaluated the PD-L1 expression and specific genomic alterations as the prognostic biomarkers for the immunotherapy of HCC. Nevertheless, the results of these studies revealed that positive expression of PD-L1 in HCC was not associated with treatment response to nivolumab or pembrolizumab. Furthermore, a lot of immune-related genes, such as TP53, were used to establish the prognostic signature of HCC (69–71). However, the prediction of single or multiple genes is not comprehensive and accurate for HCC.

## Future Directions

Recently, some preclinical studies combined chimeric antigen receptor T (CAR-T) cells with ICIs for the treatment of HCC, attaining a significant progress (72). Thus, several studies which aimed to assess the effectiveness of CAR-T cells combining with chemotherapy agents or cytokine for advanced HCC is currently ongoing (NCT02905188, NCT04093648, and NCT03198546) (73). Furthermore, the cancer vaccine in combination with ICIs may be a promising treatment option for advanced HCC in the future. The combination of these two drugs may have a synergistic anti-tumor effect that the vaccine increases the number of tumor-infiltrating effector T cells, and ICIs activate these cells (74, 75). More clinical trials to investigate this combination therapy are warranted.

Transforming growth factor beta (TGF-β) promotes tumor immune evasion by inhibiting proliferation of lymphocytes and immune factors (76). The combination of monoclonal antibodies targeting TGF-β and PD-L1 may provide a novel treatment approach because their mechanisms of action are complementary. A preclinical study indicated that the application of TGF-β and PD-L1 inhibitors could reverse the sorafenib resistance in HCC (77). In addition, a previous clinical study (NCT02699515) also confirmed the safety and preliminary efficacy of the bifunctional fusion protein in HCC (78).Many recent studies revealed that the BRAF pathway plays an important role in HCC development (79, 80). However, the inhibition of single pathway could not be sufficient to prevent HCC progression. The combination treatment of BRAF inhibitors and tyrosine kinase inhibitors (TKI) and immunotherapy made a series of exciting results (81). The preclinical study demonstrated that magnolia combined with BRAF inhibitor SB590885 inhibited the proliferation and migration of HCC cells, by targeting the ERKs/RSK2 signaling pathway (82). Furthermore, a phase I trial of Pimasertib (AS703026) confirmed the safety in HCC (83). More clinical trials are conducting.

## Author Contributions

CZ made substantial contributions to the design of the work. TO and XK drafted the manuscript. All authors contributed to the article and approved the submitted version.

## Conflict of Interest

The authors declare that the research was conducted in the absence of any commercial or financial relationships that could be construed as a potential conflict of interest.

## Publisher’s Note

All claims expressed in this article are solely those of the authors and do not necessarily represent those of their affiliated organizations, or those of the publisher, the editors and the reviewers. Any product that may be evaluated in this article, or claim that may be made by its manufacturer, is not guaranteed or endorsed by the publisher.
